# Online Health Information–Seeking Among Older Adults and Predictors of Use, Motivations, and Barriers in the Context of Healthy Aging: Cross-Sectional Study

**DOI:** 10.2196/77557

**Published:** 2026-01-06

**Authors:** Yves Bachofner, Alexander Seifert, Samin Sepahniya, Carlo Fabian

**Affiliations:** 1 Institute for Social Work and Health, School of Social Work, FHNW University of Applied Sciences and Arts, Riggenbachstrasse 16, Olten, 4600, Switzerland; 2 Institute for Integration and Participation, School of Social Work, FHNW University of Applied Sciences and Arts, Olten, Switzerland

**Keywords:** online health information seeking, healthy aging, digital competence, older adults, health literacy, aging, cross-sectional study, internet, Switzerland

## Abstract

**Background:**

Considering the rapid digital transformation, older adults are increasingly relying on online health information–seeking (OHIS) to support healthy aging. However, disparities in their digital competence levels (the ability to effectively use digital tools) and health literacy (the ability to access, understand, appraise, and apply health information) may influence engagement in OHIS.

**Objective:**

This paper examines the prevalence of OHIS among older adults in Switzerland and identifies their motivations, barriers, and predictors of use. The objective is to determine key factors that promote or hinder OHIS use among older internet users.

**Methods:**

A cross-sectional survey was conducted with 1261 internet users aged 60 years and older living in Switzerland (mean age 70.1, SD 7.3 years; 539/1261, 42.7% female). Descriptive analyses and hierarchical binary logistic regression models were used.

**Results:**

Overall, 77.6% (969/1248) of participants engaged in OHIS in their everyday lives. Subjective health status, internet use frequency, trust in online health information (OHI), and digital competence level significantly influenced OHIS use. Participants reporting good to very good health were less likely to engage in OHIS compared to those in poorer health (odds ratio [OR] 0.496, 95% CI 0.307-0.801; *P*=.004). Higher likelihood of OHIS use was associated with (almost) daily versus less frequent internet use (OR 1.550, 95% CI 1.011-2.376; *P*=.04), general trust versus distrust in OHI (OR 5.784, 95% CI 4.044-8.272; *P*<.001), and advanced versus low digital competence (OR 3.108, 95% CI 1.385-6.975; *P*=.006); health literacy was not a significant predictor of OHIS use (OR 0.912, 95% CI 0.393-2.117; *P*=.83, excellent vs deficient [reference]). Among OHIS users (n=969), the most common frequently indicated motivation for use (672/969, 69.3%) was to gain a better understanding of health conditions. Among nonusers (n=279), the most frequently indicated barriers were difficulties in assessing the credibility of information (159/279, 57%), distrust in the effectiveness of information provided (129/279, 46.2%), and concerns about dubious providers or spam (93/279, 33.3%).

**Conclusions:**

Digital competence, frequent internet use, and trust in OHI are critical for OHIS engagement among older adults. Programs to strengthen digital competencies in later life and initiatives to enhance the credibility of online health resources are essential to reduce digital disparities and support healthy aging. Notably, health literacy did not emerge as a significant factor in OHIS use, but digital competence did, suggesting that digital competence is most critical to OHIS use.

## Introduction

### Background

With a growing older population, aging presents significant health policy and societal challenges. In response, the World Health Organization’s (WHO) “Healthy Aging” [1] framework promotes well-being in later life, emphasizing that functional ability can be maintained despite health challenges. This requires physical and cognitive capacity alongside supportive physical, social, and digital environments [2]. To cope with everyday life, digital competence must increasingly be considered since digital competence not only is needed for using modern technologies but also enables digital access to health information. The rapid digital transformation, driven by modern information and communication technologies (eg, internet and smartphones), is reshaping knowledge dissemination [3]. While digital solutions enhance quality of life, health, and independence, older adults still use them less than younger groups [2,4]. This digital divide extends beyond access to include disparities in digital competence and use [5]. Indeed, many older adults face challenges due to limited digital competence. Effective digital health promotion requires both access and competencies, highlighting the critical role of digital and health literacy in using digital health services [6].

### Online Health Information Seeking Among Older Adults

Digital access is increasingly seen as a key solution for overcoming barriers to obtaining timely health information for older adults [4]. Online health information seeking (OHIS) offers a fast and convenient way to obtain qualitative health-related information but poses challenges due to limited digital competence. Older adults may struggle with navigating sources, formulating queries, and evaluating information and misinformation [7]. Despite greater health concerns, they engage in OHIS less than younger generations, partly due to age-related impairments and digital competence gaps and also because a considerable share of older adults remains offline or does not use internet-enabled devices in the first place. However, even those who use OHIS can benefit from improved access to health information, supporting healthy aging goals [8-10].

### Research Questions and Hypotheses

Despite attempts by previous studies [10] to identify the determinants of OHIS in general, the prevalence, motivations, barriers, and predictors of OHIS among older internet users (hereafter referred to as “onliners”) remain largely unclear [7,8,10]. This underscores the need for further investigation to address these gaps.

The aim of this study was to examine the prevalence, motivations, and barriers of OHIS among older onliners in Switzerland and to identify key predictors of OHIS use. Specifically, this study addressed the following research questions: (1) What proportion of onliners aged 60 years and older use OHIS? (2) What are the key determinants of OHIS use? (3) What are the motivations and barriers related to OHIS use?

Regarding the key determinants of OHIS, we proposed hypothesis 1, which assumed that sociodemographic and health-related factors influenced the likelihood of OHIS use. Specifically, we expected that female participants [7], younger individuals (aged 60‐69 years) [4,11], and participants with higher education levels [12], better financial resources [13], and urban (or intermediate) residency [14] were significantly more likely to use OHIS compared to their counterparts. Regarding health-related factors, we assumed that self-reported health status and the number of medical treatments were associated with OHIS use. While existing evidence was mixed, we expected that individuals with poorer self-reported health statuses [15] and those with more medical treatments [16] in the past year were more likely to use OHIS. Hypothesis 2 assumed that behavioral and attitudinal factors—particularly the frequency of internet use and trust in online health information (OHI)—significantly predicted OHIS use. Specifically, individuals who used the internet daily [16] and those who expressed at least some level of trust in OHI [12,17] were expected to have a greater likelihood of engaging in OHIS. Hypothesis 3 assumed that individual competencies played a critical role in OHIS use. Specifically, higher levels of digital competence [18] and health literacy [19] were expected to increase the probability of OHIS use.

## Methods

### Study Design and Participants

We conducted a cross-sectional survey within the “Regional Health Promotion in an Age-Friendly Digital World” project with individuals aged 60 years and older living in private households across Switzerland. Participants were sampled by using a stratified random sampling approach using official address data from the Swiss Federal Statistical Office in combination with an additional sampling from the private address provider AZ Direct. Surveys were carried out by Demo Scope AG, an external Swiss pooling provider.

A total of 8311 individuals were invited by mail to participate in the survey, which was available in the 3 official languages of Switzerland (German, French, and Italian). Of these, 1367 (16.4% response rate) completed the survey between June 27 and August 20, 2024, either online (computer-assisted web interviewing: n=1237) or in paper format (paper-and-pencil interviewing: n=130). Incomplete or invalid responses were excluded through rigorous data cleaning, resulting in 1325 valid questionnaires. Of these, 1261 (95.2%) respondents were classified as onliners. For the analyses, we included only the onliners because they had met the basic access requirement for OHIS use.

The questionnaire was developed based on insights from our systematic review [10] and the workshop (n=11) with older adults, family caregivers, and professionals working at the interface of age and health.

### Ethical Considerations

The Ethics Committee Northwest and Central Switzerland (Req-2023‐00727) reviewed this study and determined that it does not fall under the Human Research Act (Art. 2). The survey did not collect sensitive health-related personal data, responses were fully anonymized, and participants provided informed consent at the beginning of the survey. No compensation was provided to participants. As such, authorization from the ethics committee was not required.

### Measures

The dependent variable, OHIS, was measured via a single item: “In a typical week, how many days do you use websites for getting health-related information?” The question was adapted with minor modifications from the digital health literacy survey instrument developed by the Health Literacy Survey 2019 (HLS19) Consortium of the WHO Action Network on Measuring Population and Organizational Health Literacy [20]. Response options included “more than once per day,” “once a day,” “4‐6 days per week,” “1‐3 days per week,” “less than once per week,” “I don’t use it, but it’s interesting,” and “I don’t use it, and I’m not interested in it, either.” For analysis, responses indicating any frequency of use (“More than once per day” to “Less than once per week”) were recoded as users, while responses indicating no use were recoded as nonusers, resulting in a binary variable (use or nonuse); this approach followed established methods in prior research on OHIS [21].

To explain OHIS use, a range of sociodemographic, health-related, and individual competence factors was considered. Sociodemographic variables included sex (female or male), age group (60‐69, 70‐79, and 80‐100 years), residence location (rural, intermediate, and urban), living arrangement (living alone or with others), education level (compulsory education, secondary education, and tertiary education), and financial situation. The financial situation was assessed through a question adapted from the Swiss Survey on Income and Living Conditions, asking participants how difficult it was for their household to make ends meet with their available income, with responses categorized into “very difficult to rather difficult,” “rather simple,” and “easy to very easy” [22].

Subjective health status was measured by asking participants to rate their general health, with responses dichotomized afterward into “very poor to mediocre” and “good to very good” categories. To assess the number of medical treatments, participants were asked how often they had received medical treatment (including from general practitioners but excluding dentists) in the previous 12 months. The number of treatments ranged from 0 to 90 (mean 7.28, SD 12.68) and was dichotomized into “below the mean value (of the sample)” and “above the mean value (of the sample).” Both measures were adapted from the Swiss Federal Statistical Office Health Survey [23].

Frequency of internet use was measured by asking how often participants used the internet, with responses dichotomized into “(almost) daily use” and “less than (almost) daily use.” Trust in OHI was assessed using participants’ responses when asked how trustworthy they found health information from the internet, using a question adapted from Link and Baumann [12], with responses categorized as “rather or very trustworthy, or both trustworthy and not” versus “rather or not at all trustworthy.”

Health literacy, defined as the competencies to access, understand, appraise, and apply health information in order to make judgments and take decisions in health care, disease prevention, and health promotion, was assessed using the validated HLS19-Q12 instrument developed by the HLS19 Consortium of the WHO Action Network on Measuring Population and Organizational Health Literacy and categorized into “deficient,” “problematic,” “sufficient,” and “excellent” levels [24]. Digital competence, defined as the ability to use digital technologies in a critical, collaborative, and creative way, was measured using the DigCompSAT tool developed by Clifford et al [25], which was adapted for this study following the approach of Weinhold et al [26] and translated into German, French, and Italian by Stürz et al [27]. The overall score was divided into 4 levels: “low,” “basic,” “intermediate,” and “advanced.”

Additionally, OHIS users were asked about their motivations for and nonusers about their barriers to using OHIS, both assessed through multiple response options. The specific response categories for motivations are presented in Table S2 in Multimedia Appendix 1; categories for barriers are in Table S3 in Multimedia Appendix 1.

### Analytical Strategy

Statistical analyses were performed using SPSS (version 28; IBM Corp). Descriptive analyses comparing OHIS users (n=969) and nonusers (n=279) and their stated motivations and barriers were conducted using chi-square tests (*P* values) and Cramér *V* (effect size) to assess associations between categorical variables. To identify predictors of OHIS use, a binary logistic regression was performed, allowing for the multivariate analysis of sociodemographic, health-related, and individual competence factors.

## Results

### Sociodemographic Characteristics of the Sample

The final study sample consisted of 1261 internet users aged 60 years and older, of whom 57.3% (722/1261) were male (Table 1). A total of 52.8% (666/1261) were aged 60‐69 years, with the overall mean age being 70.1 (SD 7.3) years. Most participants lived in urban areas (718/1261, 57%), and the majority did not live alone (936/1228, 76.2%). Regarding educational attainment, 57.5% (714/1242) had completed secondary school, and 36.9% (458/1242) held a tertiary degree.

**Table 1.** Sample characteristics (N=1261) among participants aged 60 years and older who use the internet (onliners, aged 60 years and older).

	Sample, n (%)
(Registered) sex	
Female	539 (42.7)
Male	722 (57.3)
Age groups (years)	
60‐69	666 (52.8)
70‐79	438 (34.7)
80‐100	157 (12.5)
Residence location	
Rural	265 (21)
Intermediate	278 (22)
Urban	718 (57)
Living arrangement	
Living alone	292 (23.8)
Not alone	936 (76.2)
No information	33
Education	
Compulsory	70 (5.6)
Secondary school II	714 (57.5)
Tertiary level	458 (36.9)
No information	19
Financial situation	
Very difficult to rather difficult	236 (19.5)
Rather simple	334 (27.6)
Easy to very easy	639 (52.9)
No information	52
Subjective health status	
Very poor to mediocre	294 (23.5)
Good to very good	959 (76.5)
No information	8
Number of medical treatments	
Below the mean value	910 (75.9)
Above the mean value	289 (24.1)
No information	62

Financial situation was described as easy to very easy by 52.9% (639/1209), rather simple by 27.6% (n=334), and rather to very difficult by 19.5% (n=236). Most participants reported good to very good health (959/1253, 76.5%). The number of medical treatments in the previous 12 months ranged from 0 to 90; 75.9% (910/1199) were below and 24.1% (289/1199) above the sample mean (mean 7.28, SD 12.68). Table 1 provides the sample characteristics.

### Use of OHIS

Among onliners aged 60 years and older, 77.6% (969/1248) reported engaging in OHIS, while 22.4% (279/1248) did not. OHIS use was more frequent among female users (429/534, 80.3%) than male users (540/714, 75.6%), and this difference was statistically significant. Age differences were also significant, with the highest OHIS use among participants aged 60‐69 years (531/658, 80.7%), compared to 70‐79 years (320/434, 73.7%) and 80 years and older (118/156, 75.6%). Education level showed a significant association with OHIS use, with the highest use among those with tertiary education (384/455, 84.4%) compared to secondary (523/707, 74%) and compulsory schooling (49/68, 72.1%).

No significant bivariate associations were observed for residence location, living arrangement, financial situation, subjective health status, or number of medical treatments. See Table 2 for full distributions.

**Table 2.** Characteristics of online health information seeking (OHIS) users (n=969) and nonusers (n=279) among participants aged 60 years and older who use the internet (onliners, aged 60 years and older).

	OHIS user (n=969), n (%)	OHIS nonuser (n=279), n (%)	Cramér *V*^a^	*P* value
(Registered) sex			0.056	.048
Female	429 (80.3)^b^	105 (19.7)		
Male	540 (75.6)	174 (24.4)		
Age groups (years)			0.079	.02
60‐69	531 (80.7)	127 (19.3)		
70‐79	320 (73.7)	114 (26.3)		
80‐100	118 (75.6)	38 (24.4)		
Residence location			0.043	.31
Rural	201 (76.4)	62 (23.6)		
Intermediate	206 (74.9)	69 (25.1)		
Urban	562 (79.2)	148 (20.8)		
Living arrangement			0.032	.27
Living alone	219 (75.5)	71 (24.5)		
Not alone	728 (78.6)	198 (21.4)		
Education			0.123	<.001
Compulsory	49 (72.1)	19 (27.9)		
Secondary school II	523 (74)	184 (26)		
Tertiary level	384 (84.4)	71 (15.6)		
Financial situation			0.048	.25
Very difficult to rather difficult	174 (74)	61 (26)		
Rather simple	261 (78.9)	70 (21.1)		
Easy to very easy	501 (79.1)	132 (20.9)		
Subjective health status			0.047	.10
Very poor to mediocre	237 (81.2)	55 (18.8)		
Good to very good	726 (76.6)	222 (23.4)		
Number of medical treatments			0.001	.98
Below the mean value	701 (77.5)	203 (22.5)		
Above the mean value	222 (77.6)	64 (22.4)		

a Reported Cramér *V* values with corresponding *P* values indicate the strength and significance of group differences.

b Percentages are calculated within subgroups (users vs nonusers).

### Predictors of OHIS

To identify significant predictors of OHIS, 3 hierarchical binary logistic regression models were conducted. These models sequentially examined the effects of sociodemographic (sex, age, education, financial situation, residence location, and living arrangement) and health-related (subjective health and number of medical treatments) factors (model 1), internet use and trust in OHI (model 2), and individual health literacy and digital competence (model 3; Table 3).

**Table 3.** Binary logistic regression^a^ models predicting online health information seeking (OHIS) use among onliners aged 60 years and older (n=1043) across sociodemographic and health-related factors, internet use and online health information (OHI) trust, and individual competence^b^.

Predictors	Model 1: sociodemographic and health-related factors		Model 2: model 1 factors plus internet use and OHI trust		Model 3: model 2 factors plus digital competence and health literacy	
	OR^c^ (95% CI)	*P* value	OR (95% CI)	*P *value	OR (95% CI)	*P* value
(Registered) sex						
Male	Reference	Reference	Reference	Reference	Reference	Reference
Female	1.369 (0.981-1.912)	.06	1.295 (0.902-1.860)	.16	1.409 (0.972-2.043)	.07
Age groups (years)						
60‐69	Reference	Reference	Reference	Reference	Reference	Reference
70‐79	0.696 (0.498-0.972)	.03	0.757 (0.527-1.088)	.13	0.782 (0.540-1.132)	.19
80‐100	0.989 (0.424-1.122)	.13	0.790 (0.465-1.343)	.38	0.884 (0.512-1.524)	.66
Residence location						
Rural	Reference	Reference	Reference	Reference	Reference	Reference
Intermediate	1.020 (0.641-1.621)	.94	1.032 (0.625-1.706)	.90	1.010 (0.607-1.681)	.97
Urban	1.094 (0.740-1.618)	.65	0.998 (0.652-1.528)	.99	0.983 (0.638-1.514)	.94
Living arrangement						
Living alone	Reference	Reference	Reference	Reference	Reference	Reference
Not alone	1.271 (0.876-1.844)	.21	1.325 (0.886-1.982)	.17	1.319 (0.877-1.982)	.18
Education						
Compulsory	Reference	Reference	Reference	Reference	Reference	Reference
Secondary school II	1.115 (0.566-2.196)	.75	0.943 (0.442-2.009)	.88	0.748 (0.346-1.619)	.46
Tertiary level	1.994 (0.964-4.1259)	.06	1.353 (0.601-3.050)	.47	0.996 (0.432-2.293)	.99
Financial situation						
Very difficult to rather difficult	Reference	Reference	Reference	Reference	Reference	Reference
Rather simple	1.356 (0.860-2.138)	.19	1.332 (0.813-2.182)	.26	1.310 (0.794-2.162)	.29
Easy to very easy	1.394 (0.917-2.120)	.12	1.381 (0.873-2.186)	.17	1.322 (0.824-2.121)	.25
Subjective health status						
Very poor to mediocre	Reference	Reference	Reference	Reference	Reference	Reference
Good to very good	0.537 (0.344-0.837)	.006	0.505 (0.315-0.811)	.005	0.496 (0.307-0.801)	.004
Number of medical treatments						
Below the mean value	Reference	Reference	Reference	Reference	Reference	Reference
Above the mean value	0.780 (0.522-1.167)	.23	0.753 (0.488-1.162)	.20	0.774 (0.501-1.198)	.25
Internet use						
Less than (almost) daily	—^d^	—	Reference	Reference	Reference	Reference
(Almost) daily internet use	—	—	1.970 (1.321-2.937)	<.001	1.550 (1.011-2.376)	.04
Trust in OHI						
Rather or not at all trustworthy	—	—	Reference	Reference	Reference	Reference
OHI are rather or very trustworthy, or both trustworthy and not	—	—	6.026 (4.252-8.542)	<.001	5.784 (4.044-8.272)	<.001
Health literacy (HLS19-Q12)						
Deficient	—	—	—	—	Reference	Reference
Problematic	—	—	—	—	0.733 (0.400-1.346)	.32
Sufficient	—	—	—	—	0.669 (0.349-1.282)	.23
Excellent	—	—	—	—	0.912 (0.393-2.117)	.83
Digital competence (DigCompSAT)						
Low	—	—	—	—	Reference	Reference
Basic	—	—	—	—	1.811 (0.990-3.316)	.05
Intermediate	—	—	—	—	2.660 (1.467-4.824)	.001
Advanced	—	—	—	—	3.108 (1.385-6.975)	.006

a Dependent variable: user OHIS=1, nonuser OHIS=0. For detailed statistical values (CIs), please refer to Table S1 in Multimedia Appendix 1.

b Model fit: model 1: Nagelkerke *R*2=0.045; *χ*2_12_=30.2; *P*=.003; model 2: Nagelkerke *R*2=0.217; *χ*2_14_=154.3; *P*<.001; and model 3: Nagelkerke *R*2=0.234; *χ*2_20_=167.4; *P*<.001.

c OR: odds ratio.

d The predictor was not included in the respective model.

Model 1 (Nagelkerke *R*^2^=0.045; *χ*^2^_12_=30.2; *P*=.003) was statistically significant and revealed that only age was a significant predictor within the sociodemographic variables. Participants aged 70‐79 years were significantly less likely to use OHIS compared to those aged 60‐69 years (odds ratio [OR] 0.696, 95% CI 0.498-0.972; *P*=.03). Notably, no significant difference was observed between participants aged 80‐100 years and those aged 60‐69 years (OR 0.989, 95% CI 0.424-1.122; *P*=.13). In contrast, other sociodemographic factors that were significant in the bivariate analysis—sex and education level—did not retain significance in the multivariate model. Besides age, subjective health was also a significant predictor. Participants who rated their health as good to very good were less likely to use OHIS compared to those with poor to mediocre health (OR 0.537, 95% CI 0.344-0.837; *P*=.006). Conversely, the number of medical treatments in the previous year showed no significant association with OHIS engagement (OR 0.780, 95% CI 0.522-1.167; *P*=.23). These results provide mixed support for hypothesis 1.

Model 2 (Nagelkerke *R*^2^=0.217; *χ*^2^_14_=154.3; *P<*.001) introduced internet use frequency and trust in OHI as predictors. The analysis revealed that both factors were significant predictors of OHIS use, providing full support for hypothesis 2. Participants who reported using the internet (almost) daily were nearly twice as likely to use OHIS compared to those who used it less frequently (OR 1.970, 95% CI 1.321‐2.937; *P*<.001). Additionally, participants who perceived OHI as rather or very trustworthy, or both trustworthy and not, were over 6 times more likely to use OHIS than those who distrusted OHI (OR 6.026, 95% CI 4.252‐8.542; *P*<.001). Notably, the previously significant effect of age became nonsignificant after including these 2 model 2 variables (OR 0.757, 95% CI 0.527-1.088; *P*=.13).

Model 3 (Nagelkerke *R*^2^=0.234; *χ*^2^_20_=167.4; *P<*.001) added health literacy and digital competence to the analysis. Compared to adults with low digital competence levels, those with intermediate competence were more than twice as likely to use OHIS (OR 2.660, 95% CI 1.467‐4.824; *P*=.001), and those with advanced competence were over 3 times more likely (OR 3.108, 95% CI 1.385‐6.975; *P*=.006) to use OHIS. In contrast, health literacy was not a significant predictor. Additionally, subjective health status, daily internet use, and trust in OHI continued to be significant predictors in model 3.

The model’s explanatory power increased with each step, as indicated by the rising Nagelkerke *R*², from 0.045 in model 1 to 0.234 in model 3. This progression highlights how the inclusion of internet use, trust in OHI, and digital competence substantially improved the model’s ability to predict OHIS use.

### Motivations for OHIS

Among the 969 OHIS users, the most commonly indicated reason for use was to gain a better understanding of certain health conditions or illnesses (672/969, 69.3%), followed by learning about medications and their possible side effects (538/969, 55.5%) and searching for treatment options or therapies for specific health problems (528/969; 54.5%; Table 4). Additionally, searching for alternative or complementary medical approaches (424/969, 43.8%) and seeking information out of general interest (402/969, 41.5%) were notable motivations. Fewer participants indicated using OHIS to obtain a second opinion (180/969, 18.6%) or for other reasons (9/969, 0.9%; eg, assisting family members and searching for information when health professionals are unavailable).

**Table 4.** Motivations for engaging in online health information seeking (OHIS) among OHIS users (n=969) within the onliner population (aged 60 years and older), including chi-square tests for sex and age differences^a^ ^b^.

Motivation (multiple response options)	Total, n (%)	Male, n (%)	Female, n (%)	Chi-square test for differences in sex, *P* value	60‐69 years, n (%)	70‐79 years, n (%)	80‐100 years, n (%)	Chi-square test for differences in age, *P* value
Understanding health conditions	672 (69.3)	372 (68.9)	300 (69.9)	.73	370 (69.7)	216 (67.5)	86 (72.9)	.54
Medications and side effects	538 (55.5)	284 (52.6)	254 (59.2)	.04	268 (50.5)	197 (61.6)	73 (61.9)	.002
Treatment options or therapies	528 (54.5)	262 (48.5)	266 (62)	<.001	274 (51.6)	190 (59.4)	64 (54.2)	.09
Alternative or complementary medicine	424 (43.8)	192 (35.6)	232 (54.1)	<.001	239 (45)	139 (43.4)	46 (39)	.49
Just out of interest	402 (41.5)	233 (43.1)	169 (39.4)	.24	241 (45.4)	112 (35)	49 (41.5)	.01
Second opinion	180 (18.6)	118 (21.9)	62 (14.5)	.003	89 (16.8)	63 (19.7)	28 (23.7)	.18
Other reasons	9 (0.9)	8 (1.5)	1 (0.2)	N/A^c^	4 (0.8)	3 (0.9)	2 (1.7)	N/A

a Detailed effect sizes (Cramér *V*) and full answer options from the survey are reported in Table S2 in Multimedia Appendix 1.

b Sorted by total.

c N/A indicates that no calculation was performed because cells had a frequency of fewer than 5.

Sex differences were significant for several motivations. Female participants were more likely than male participants to search for information on treatment options or therapies (266/429, 62% vs 262/540, 48.5%), alternative or complementary medical approaches (232/429, 54.1% vs 192/540, 35.6%), and medications and side effects (254/429, 59.2% vs 284/540, 52.6%). Conversely, male participants were more inclined to search for a second opinion (118/540, 21.9% vs 62/429, 14.5%).

Significant age-related differences also emerged. Older participants, particularly those aged 70‐79 (197/320, 61.6%) and 80‐100 years (73/118, 61.9%), were more likely to seek information about medications and side effects compared to the 60‐ to 69-year age group (268/531, 50.5%). In contrast, younger participants (aged 60-69 years) were more likely to search for OHI out of general interest (241/531, 45.4%) than older groups.

### Barriers to OHIS

The most commonly indicated barrier to use among OHIS nonusers was difficulty assessing the credibility of information (159/279, 57%), followed by distrust in the effectiveness of the information provided (129/279, 46.2%), concerns about dubious providers or the risk of spam and advertising (93/279, 33.3%), lack of experience with searching for information on the internet (87/279, 31.2%), and challenges related to technical or difficult-to-understand language in health information (46/279, 16.5%; Table 5). Fewer participants indicated barriers such as lack of support in using digital services (20/279, 7.2%), negative past experiences with online searches (17/279, 6.1%), physical limitations when using digital devices (10/279, 3.6%), and other reasons (51/279, 18.3%, eg, outdated or unclear publication dates and lack of personal interest in health information). Sex- or age-related differences did not attain statistical significance for any of the barriers.

**Table 5.** Barriers to engaging in online health information seeking (OHIS) among OHIS nonusers (n=279) within the onliner population (60 years and older), including chi-square tests for sex and age differences^a^ ^b^.

Barriers (multiple response options)	Total, n (%)	Male, n (%)	Female, n (%)	Chi-square test for differences in sex, *P* value	60‐69 years, n (%)	70‐79 years, n (%)	80‐100 years, n (%)	Chi-square test for differences in age, *P* value
Credibility	159 (57)	96 (55.2)	63 (60)	.43	71 (55.9)	64 (56.1)	24 (63.2)	.71
Distrust	129 (46.2)	84 (48.3)	45 (42.9)	.38	61 (48)	51 (44.7)	17 (44.7)	.86
Dubious offers	93 (33.3)	60 (34.5)	33 (31.4)	.60	47 (37)	39 (34.2)	7 (18.4)	.10
Lack of experience	87 (31.2)	56 (32.2)	31 (29.5)	.64	32 (25.2)	38 (33.3)	17 (44.7)	.06
Technical language	46 (16.5)	31 (17.8)	15 (14.3)	.44	20 (15.7)	20 (17.5)	6 (15.8)	.93
Lack of support	20 (7.2)	12 (6.9)	8 (7.6)	.82	8 (6.3)	8 (7)	4 (10.5)	N/A^c^
Negative experiences	17 (6.1)	12 (6.9)	5 (4.8)	.47	10 (7.9)	4 (3.5)	3 (7.9)	N/A
Physical limitations	10 (3.6)	4 (2.3)	6 (5.7)	N/A	3 (2.4)	5 (4.4)	2 (5.3)	.59
Other reasons	51 (18.3)	30 (17.2)	21 (20)	.56	20 (15.7)	23 (20.2)	8 (21.1)	.60

a Detailed effect sizes (Cramér *V*) and full answer options from the survey are reported in Table S3 in Multimedia Appendix 1.

b Sorted by total.

c N/A indicates that no calculation was performed because cells had a frequency of fewer than 5.

## Discussion

### Principal Findings

The study findings revealed that OHIS occurred widely among older adults in this demographic, with 77.6% (n=969) of older onliners using OHIS. This aligns with prior research demonstrating high engagement with digital health resources among older adults [28]. Notably, no significant difference in OHIS engagement was found between individuals aged 80‐100 years and the younger age groups, although a drop in use was observed in the 70‐ to 79-year age group compared to the 60‐ to 69-year group. This suggests that the oldest age group may have adapted to digital tools similarly to younger older adults [4]. One potential explanation for this negligible discrepancy may be that the younger age group (60-69 years) was more inclined to experiment with technology and explore digital tools, consequently resulting in higher OHIS use. In contrast, the oldest group (80-100 years) may be more predisposed to seek information online for health reasons [29]. Furthermore, this study revealed a marginally elevated propensity among female participants to use OHIS, aligning with the extant literature suggesting that female participants exhibit a heightened propensity to proactively seek health-related information [7].

Education emerged as a significant predictor of OHIS use. Individuals with tertiary education were more likely to seek health information online, supporting the theory of the digital divide, where higher education correlates with better digital competence and greater access to online resources [5].

In the multivariate analysis, the effects of education, sex, and age lost statistical significance. This suggests that, while these sociodemographic factors may initially appear associated with OHIS use, their explanatory power diminishes when health, behavioral, and competence-related variables, such as subjective health status, digital competence, and trust in OHI, are considered. This pattern aligns with previous findings that highlight the centrality of these more proximal determinants [21]. This highlights the importance of broader structural and individual determinants in shaping OHIS use.

Markedly, individuals with poorer self-reported health statuses were more likely to use OHIS, supporting findings that health concerns drive proactive information seeking [30]. However, the number of medical treatments was not associated with OHIS engagement, suggesting that health care use alone does not motivate OHIS. Instead, sufficient information from health care providers may reduce the need for additional online searches, while other providers may encourage OHIS use [16].

The predictive role of digital competence was shown within our analyses; people with higher levels of digital competence were more often within the group of OHIS users. A higher level of digital competence can facilitate the ability to search for OHI, while those with low competence levels remained disengaged, despite internet access, underscoring that mere access is insufficient for effective use [6,18].

Moreover, regular use of the internet also predicted OHIS use and can be regarded as a behavioral indicator of technological familiarity, thereby further supporting the application of OHIS. However, digital competence encompasses a more extensive ability to effectively engage with digital tools across various contexts.

Contrary to the findings of other studies, health literacy was not a significant predictor of OHIS use in this research [7,19]. This suggests that, while individuals with lower health literacy may face challenges in comprehending and critically evaluating health information, these difficulties do not necessarily prevent them from OHIS engagement. The ease of access and widespread availability of OHI may encourage use regardless of comprehension levels. However, this raises concerns about the potential risk of misinterpretation or reliance on misleading information, particularly among those with lower health literacy levels. This highlights that OHIS primarily reflects the act of searching rather than the quality of comprehension or application, a finding consistent with Wang et al [21], who emphasized that instrumental factors, such as utility and trust, are far stronger predictors of OHIS than psychological or cognitive abilities related to processing health information. As a result, individuals with lower health literacy may still use OHIS without necessarily deriving meaningful health benefits. This underscores the need for integrated strategies that strengthen both digital competence and health literacy to ensure that access to information translates into informed decision-making and improved health outcomes.

Of the variables included, trust in OHI proved to be the strongest predictor of OHIS use. Participants who perceived OHI as trustworthy were significantly more likely to engage in OHIS, underscoring the central role that perceived credibility plays in online health behaviors. This finding aligns with prior research, which has consistently shown that trust is a key determinant in digital health use [7,21,31,32].

Conversely, a lack of trust in OHI was among the barriers most frequently cited by nonusers. This distrust often stems from concerns about misinformation, unreliable sources, and commercial influences [31]. In line with previous studies, respondents expressed apprehension regarding the credibility of online health resources, which aligns with findings from Sbaffi and Rowley [33], who emphasized that website design, intrusive advertisements, and complex language negatively affect the perceived trustworthiness of OHI.

Importantly, sex and age differences indicated distinct information-seeking patterns, with female participants more focused on treatment-related topics and alternative medicine and male participants more likely to seek second opinions, while younger participants demonstrated a broader, more general interest in health-related content compared to older age groups. Therefore, digital health information should always consider the different audiences and, if necessary, tailor its content to specific audiences.

### Implications for Practice and Policy

Enhancing digital competence through targeted training could improve OHIS use, especially among older adults with low digital competence levels [7]. Public health campaigns should build trust in OHI by promoting credible and user-friendly digital health platforms. Addressing individual capabilities and improving the quality of digital health information can help bridge gaps in OHIS use [30]. As highlighted by Jacob et al [34], the effectiveness of digital health interventions depends not only on providing information but also on ensuring user trust through privacy, security, and credibility. For offline individuals, the challenge lies in gaining access to digital resources. Expanding digital infrastructures and providing accessible training are essential first steps toward enabling digital engagement [10]. However, reliable offline health information (eg, flyers and brochures from government health organizations) must continue to be available to meet the needs of those who do not engage with digital platforms.

### Limitations

This study has several limitations. As it focuses on Switzerland alone, the generalizability of our findings to other contexts may be limited. The cross-sectional design prevents time comparisons and, therefore, causal conclusions about factors influencing OHIS use. Hence, future longitudinal studies should investigate factors that influence changes in OHIS use over time. Self-reported data, such as subjective health, may introduce recall or social desirability bias, potentially affecting the accuracy of responses. Additionally, the content and quality of the accessed health information were not assessed, limiting insights into the variance of the individual user profiles.

### Conclusions

This paper highlights the significant correlation of subjective health status, digital competence, daily internet use, and trust in OHI with OHIS use among older adults. Health literacy and sociodemographic characteristics showed no significant correlation when examined alongside other factors. Addressing digital competence and enhancing trust in OHI are essential for reducing digital inequalities and empowering older adults to manage their health more actively, thereby promoting healthy aging.

## Supplementary material

10.2196/77557 Multimedia Appendix 1 Supplemental tables for detailed statistical values.

## Abbreviations

HLS19: Health Literacy Survey 2019
OHI: online health information
OHIS: online health information seeking
OR: odds ratio
WHO: World Health Organization
