# Supplementary material for: Online Health Information–Seeking Among Older Adults and Predictors of Use, Motivations, and Barriers in the Context of Healthy Aging: Cross-Sectional Study
Source: Online J Public Health Inform. 2026 Jan 6;18:e77557. doi: 10.2196/77557 (PMC12772427; doi:10.2196/77557)
Supplement: Multimedia Appendix 1 [file ojphi-v18-e77557-s001.docx]

Table S1: Binary logistic regression^a^ models predicting OHIS usage among Onliners aged 60+ (n = 1043) across sociodemographic and health - related factors, Internet usage and OHI trust, and individual competence.

| Predictors | | Model 1^b^:  Socio - demographic and health - related factors | | | Model 2^c^:  Internet usage and OHI trust dimension | | | Model 3^d^:  Individual competences | | |
| --- | --- | --- | --- | --- | --- | --- | --- | --- | --- | --- |
|  |  | OR | 95% CI | *P* | OR | 95% CI | *P* | OR | 95% CI | *P* |
| (registered) Gender | Male | Ref | Ref | Ref | Ref | Ref | Ref | Ref | Ref | Ref |
|  | Female | 1.369 | .981 - 1.912 | .065 | 1.295 | .902 - 1.860 | .161 | 1.409 | .972 - 2.043 | .070 |
| Age groups | 60 - 69 | Ref | Ref | Ref | Ref | Ref | Ref | Ref | Ref | Ref |
|  | 70 - 79 | .696 | .498 - .972 | **.033** | .757 | .527 - 1.088 | .132 | .782 | .540 - 1.132 | .192 |
|  | 80 - 100 | .989 | .424 - 1.122 | .134 | .790 | .465 - 1.343 | .384 | .884 | .512 - 1.524 | .657 |
| Residence location | Rural | Ref | Ref | Ref | Ref | Ref | Ref | Ref | Ref | Ref |
|  | Intermediate | 1.020 | .641 - 1.621 | .935 | 1.032 | .625 - 1.706 | .901 | 1.010 | .607 - 1.681 | .970 |
|  | Urban | 1.094 | .740 - 1.618 | .652 | .998 | .652 - 1.528 | .992 | .983 | .638 - 1.514 | .937 |
| Living Arrangement | Living alone | Ref | Ref | Ref | Ref | Ref | Ref | Ref | Ref | Ref |
|  | Not alone | 1.271 | .876 - 1.844 | .206 | 1.325 | .886 - 1.982 | .171 | 1.319 | .877 - 1.982 | .183 |
| Education | Compulsory | Ref | Ref | Ref | Ref | Ref | Ref | Ref | Ref | Ref |
|  | Secondary school II | 1.115 | .566 - 2.196 | .754 | .943 | .442 - 2.009 | .878 | .748 | .346 - 1.619 | .461 |
|  | Tertiary level | 1.994 | .964 - 4.125 | .063 | 1.353 | .601 - 3.050 | .465 | .996 | .432 - 2.293 | .992 |
| Financial situation | Very difficult to rather difficult | Ref | Ref | Ref | Ref | Ref | Ref | Ref | Ref | Ref |
|  | Rather simple | 1.356 | .860 - 2.138 | .190 | 1.332 | .813 - 2.182 | .255 | 1.310 | .794 - 2.162 | .291 |
|  | Easy to very easy | 1.394 | .917 - 2.120 | .120 | 1.381 | .873 - 2.186 | .168 | 1.322 | .824 - 2.121 | .248 |
| Subjective health status | Very poor to mediocre | Ref | Ref | Ref | Ref | Ref | Ref | Ref | Ref | Ref |
|  | Good to very good | .537 | .344 - .837 | **.006** | .505 | .315 - .811 | **.005** | .496 | .307 - .801 | **.004** |
| Number of medical treatments | Below the mean value | Ref | Ref | Ref | Ref | Ref | Ref | Ref | Ref | Ref |
|  | Above the mean value | .780 | .522 - 1.167 | .227 | .753 | .488 - 1.162 | .200 | .774 | .501 - 1.198 | .251 |
| Internet use | Less than (almost) daily | —^e^ | — | — | Ref | Ref | Ref | Ref | Ref | Ref |
|  | (Almost) daily internet use | — | — | — | 1.970 | 1.321 - 2.937 | **<.001** | 1.550 | 1.011 - 2.376 | **.044** |
| Trust in OHI | Rather or not at all trustworthy (Ref.) | — | — | — | Ref | Ref | Ref | Ref | Ref | Ref |
|  | OHI are rather or very trustworthy, or both trustworthy and not | — | — | — | 6.026 | 4.252 - 8.542 | **<.001** | 5.784 | 4.044 - 8.272 | **<.001** |
| Health literacy  (HLS_19_ - Q12) | Deficient (Ref.) | — | — | — | — | — | — | Ref | Ref | Ref |
|  | Problematic | — | — | — | — | — | — | .733 | .400 - 1.346 | .316 |
|  | Sufficient | — | — | — | — | — | — | .669 | .349 - 1.282 | .226 |
|  | Excellent | — | — | — | — | — | — | .912 | .393 - 2.117 | .830 |
| Digital competence (DigCompSAT) | Low (Ref.) | — | — | — | — | — | — | Ref | Ref | Ref |
|  | Basic | — | — | — | — | — | — | 1.811 | .990 - 3.316 | .054 |
|  | Intermediate | — | — | — | — | — | — | 2.660 | 1.467 - 4.824 | **.001** |
|  | Advanced | — | — | — | — | — | — | 3.108 | 1.385 - 6.975 | **.006** |

^a^Dependent variable: user OHIS = 1, non - user OHIS = 0; significant differences in bold; OR, Odds Ratio; Ref, reference category.

^b^Model 1 = Nagelkerkes R^2^ = .045; Cox & Snell R^2^ = .029; χ²(12) = 30.169; *P* = .003

^c^Model 2 = Nagelkerkes R^2^ = .217; Cox & Snell R^2^ = .138; χ²(14) = 154.335; *P* < .001

^d^Model 3 = Nagelkerkes R^2^ = .234; Cox & Snell R^2^ = .148; χ²(20) = 167.434; *P* < .001

^e^ “—” indicates that the predictor was not included in the respective model.

Table S2: Motivations for engaging in OHIS among OHIS user (n = 969) within the Onliner population (60+), including Cramér’s V Statistics for gender and age differences. Sorted by total.

| Multiple response options | Total  n (%) | Male  n (%) | Female  n (%) | Cramér’s V (*P*) | 60 - 69  n (%) | 70 - 79  n (%) | 80 - 100  n (%) | Cramér’s V (*P*) |
| --- | --- | --- | --- | --- | --- | --- | --- | --- |
| Improving one's understanding of certain health conditions or illnesses | 672 (69.3) | 372 (68.9) | 300 (69.9) | .011 (.727) | 370 (69.7) | 216 (67.5) | 86 (72.9) | .036 (.539) |
| Obtaining information about medications and their possible side effects | 538 (55.5) | 284 (52.6) | 254 (59.2) | .066 **(.040**) | 268 (50.5) | 197 (61.6) | 73 (61.9) | .112 (**.002**) |
| Search for treatment options or therapies for specific health problems | 528 (54.5) | 262 (48.5) | 266 (62.0) | .135 **(<.001**) | 274 (51.6) | 190 (59.4) | 64 (54.2) | .071 (.088) |
| Search for alternative or complementary medical approaches | 424 (43.8) | 192 (35.6) | 232 (54.1) | .185 **(<.001**) | 239 (45.0) | 139 (43.4) | 46 (39.0) | .039 (.486) |
| Just out of interest | 402 (41.5) | 233 (43.1) | 169 (39.4) | .038 (.239) | 241 (45.4) | 112 (35.0) | 49 (41.5) | .096 (.**012**) |
| Obtaining a second opinion | 180 (18.6) | 118 (21.9) | 62 (14.5) | .095 **(.003**) | 89 (16.8) | 63 (19.7) | 28 (23.7) | .060 (.175) |
| Other reasons | 9 (.9) | 8 (1.5) | 1 (0.2) | NA^a^ | 4 (0.8) | 3 (0.9) | 2 (1.7) | NA |

^a^NA: No calculation was performed because cells had a frequency of fewer than five.

Table S3: Barriers to engaging in OHIS among OHIS non-user (n = 279) within the Onliner population (60+), including Cramér’s V Statistics for gender and age differences. Sorted by total.

| Multiple response options | Total  n (%) | Male  n (%) | Female  n (%) | Cramér’s V (*P*) | 60 - 69  n (%) | 70 - 79  n (%) | 80 - 100  n (%) | Cramér’s V (*P*) |
| --- | --- | --- | --- | --- | --- | --- | --- | --- |
| Credibility: difficulties in assessing the credibility of information | 159 (57.0) | 96 (55.2) | 63 (60.0) | .047 (.430) | 71 (55.9) | 64 (56.1) | 24 (63.2) | .050 (.710) |
| Distrust: Disbelief in the effectiveness of the health information provided | 129 (46.2) | 84 (48.3) | 45 (42.9) | .053 (.379) | 61 (48.0) | 51 (44.7) | 17 (44.7) | .033 (.860) |
| Dubious offers: dubious providers, risk of spam (advertising) | 93 (33.3) | 60 (34.5) | 33 (31.4) | .031 (.600) | 47 (37.0) | 39 (34.2) | 7 (18.4) | .129 (.100) |
| Lack of experience: little experience with searching for information on the internet | 87 (31.2) | 56 (32.2) | 31 (29.5) | .028 (.642) | 32 (25.2) | 38 (33.3) | 17 (44.7) | .141 (.060) |
| Technical language: Complicated or difficult - to - understand language in texts/information | 46 (16.5) | 31 (17.8) | 15 (14.3) | .046 (.441) | 20 (15.7) | 20 (17.5) | 6 (15.8) | .024 (.925) |
| Lack of support: No support in using the digital services | 20 (7.2) | 12 (6.9) | 8 (7.6) | .014 (.821) | 8 (6.3) | 8 (7.0) | 4 (10.5) | NA^a^ |
| Negative experiences: Negative experiences with searching for information on the internet | 17 (6.1) | 12 (6.9) | 5 (4.8) | .043 (.470) | 10 (7.9) | 4 (3.5) | 3 (7.9) | NA |
| Physical limitations: difficulties in using digital services | 10 (3.6) | 4 (2.3) | 6 (5.7) | NA | 3 (2.4) | 5 (4.4) | 2 (5.3) | .062 (.585) |
| Other reasons | 51 (18.3) | 30 (17.2) | 21 (20.0) | .035 (.564) | 20 (15.7) | 23 (20.2) | 8 (21.1) | .060 (.602) |

^a^NA: No calculation was performed because cells had a frequency of fewer than five.
